# Incidental multiple myeloma in a patient with neuroretinitis: a case report

**DOI:** 10.1186/s12886-023-03220-9

**Published:** 2023-12-04

**Authors:** Taesung Joo, Tae Gi Kim, Sang Woong Moon

**Affiliations:** Department of Ophthalmology, Kyung Hee University Hospital at Gangdong, Kyung Hee University School of Medicine, 892, Dongnam-ro, Gangdong-gu, Seoul, 05278 Republic of Korea

**Keywords:** Neuroretinitis, Multiple Myeloma, Macular star

## Abstract

**Background:**

Neuroretinitis is classically defined as a clinical triad of unilateral, painless vision loss, accompanied by optic disc edema and characteristic macular star formation. The causes of neuroretinitis can be categorized as infectious, non-infectious, and idiopathic, therefore differential diagnosis and careful evaluation are required, owing to the various etiologies and masqueraders.

**Case presentation:**

A 54-year-old woman presented to the clinic with blurred vision in both eyes. A complete ophthalmic examination revealed optic disc edema with blurred margins and macular exudates, intraretinal edema in the temporal peripapillary area, and subretinal fluid with neurosensory retinal detachment in the macular area. Systemic laboratory investigations showed no signs of infection or inflammation. However, bone marrow suppression was suspected based on the results of the complete blood count test, and the patient was diagnosed with multiple myeloma.

**Conclusion:**

Although neuroretinitis is rarely accompanied by hematological malignancy, it is important to be mindful of the latter because ophthalmic manifestations are a common feature of hematological malignancies and lesions occur in nearly every ocular structure.

## Background

Neuroretinitis is classically defined as a clinical triad of unilateral, painless vision loss, accompanied by optic disc edema and characteristic macular star formation. However, the clinical triad may not always be present in its entirety, especially in the early stages of the disease [1]. Additionally, dyschromatopsia, relative afferent pupillary defect, and visual field abnormalities, most commonly cecocentral/central scotoma, may also occur, although anterior chamber reaction is very rare [2].

The etiology of neuroretinitis is wide-ranging and should be determined early in the evaluation process to guide the treatment approach. Neuroretinitis may be categorized as infectious or non-infectious based on its etiology [1].

Among the causes of infectious neuroretinitis, cat scratch disease caused by Bartonella henselae has consistently been shown to be the most common [3]. Other infectious causes include involving a wide range of organisms, such as viruses, fungi, and parasites. Non-infectious neuroretinitis may be caused by underlying systemic inflammatory disorders, such as sarcoidosis; however, they are often determined to be idiopathic. The diagnosis of idiopathic neuroretinitis should be considered in cases where no infectious or non-infectious causes can be identified [1].

Due to its varied etiology, a robust differential diagnosis and careful evaluation are needed. In the present case, we found no evidence of infection or other systemic abnormalities on several tests, including laboratory tests. However, a patient with neuroretinitis was referred to the Department of Hemato Oncology upon suspicion of bone marrow suppression based on the complete blood count, and diagnosed with multiple myeloma. To the best of our knowledge, no study has reported on the association between multiple myeloma and neuroretinitis, making this is the first case report of neuroretinitis related to multiple myeloma.

## Case presentation

A 54-year-old woman visited our hospital with a complaint of blurred vision in both eyes for 1 month. Six weeks ago, she had flu-like symptoms, which lasted for approximately 1 week. There was no other past medical history, travel history, or contact with animals such as cats or dogs.

**Fig. 1:**
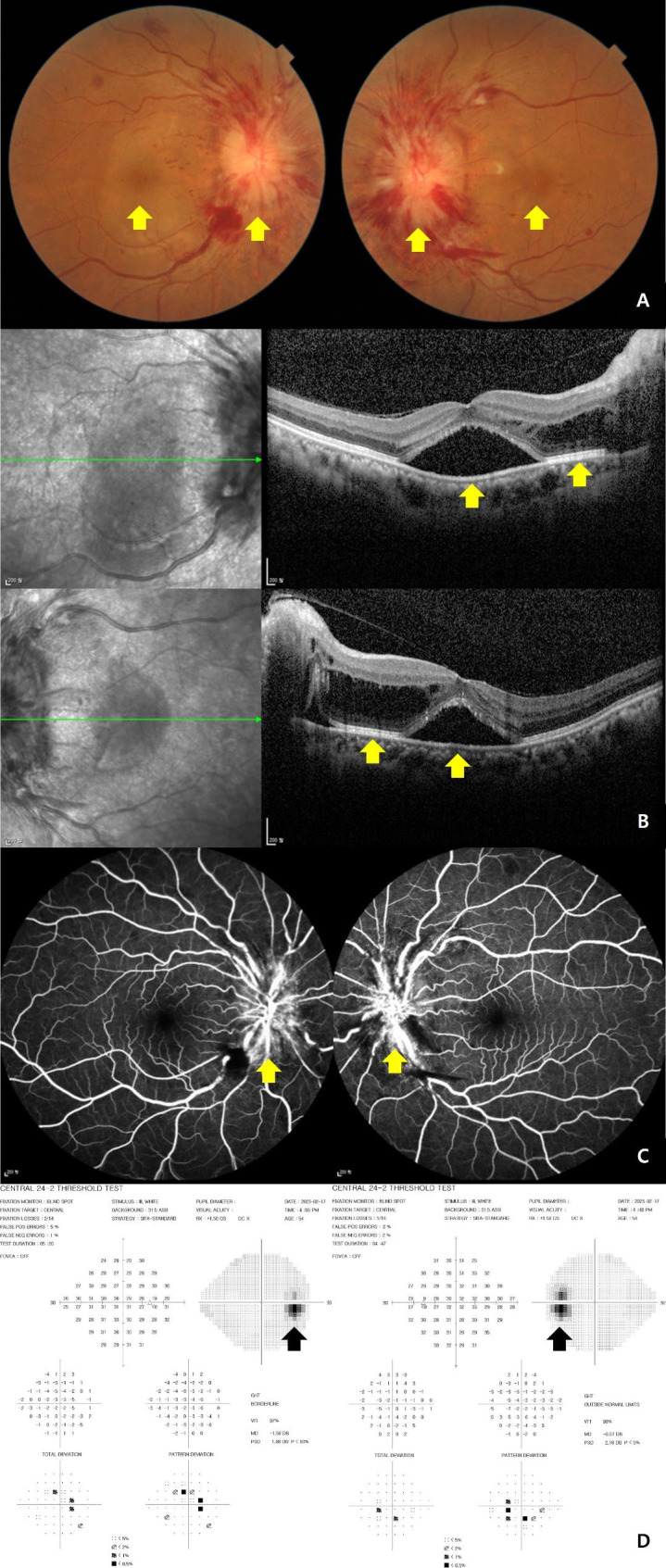
Initial images from a 54-year-old woman with idiopathic neuroretinitis in both eyes **(A)** Color fundus photography. **(B)** Spectral domain optical coherence tomography (SD-OCT). **(C)** Fluorescein angiography (FA). **(D)** The Humphrey visual field 24 – 2 test

The best-corrected visual acuity was 20/63 in the right eye and 20/32 in the left eye. Anterior segment examination yielded unremarkable results, and the intraocular pressure was 18 mmHg in both eyes. Ocular movement and the pupils were normal without diplopia or relative afferent pupillary defect, and the patient did not complain of ocular pain. The results of the color vision test using Ishihara’s plates were normal for both eyes. Fundus photography revealed swollen optic discs with blurred margins and macular exudates in both eyes (Fig. 1A). Spectral-domain optical coherence tomography revealed intraretinal edema in the temporal peripapillary area and subretinal fluid with neurosensory retinal detachment in the macular area (Fig. 1B). Fluorescein angiography showed leakage at the disc with no evidence of retinal vasculitis or late staining of the venous walls (Fig. 1C). The Humphrey visual field 24 – 2 test showed only mild blind spot enlargement without scotoma or defects in either eye (Fig. 1D). Blood pressure was measured to check for malignant hypertension, and the result was 136/82, which is not malignant hypertension. Brain and orbital magnetic resonance imaging, which were performed to confirm the presence/absence of optic nerve diseases such as optic neuritis and papilledema, showed no definitive findings of these diseases.

A systemic laboratory workup, including complete blood count, serum biochemistry, and serologic and immunologic tests for syphilis, tuberculosis, influenza, varicella, toxoplasmosis, toxocariasis, human immunodeficiency virus (HIV), cytomegalovirus (CMV), anti-aquaporin 4 Ab, angiotensin converting enzyme (ACE), anti-nuclear antibody (ANA)&anti-neutrophil cytoplasmic antibody (ANCA), rheumatoid factor (RF), and anti-CCP, was performed to rule out underlying conditions that may cause infectious and inflammatory disorders. The test results revealed no evidence of infection or inflammation. However, bone marrow suppression was suspected on the complete blood count test: low red blood cells, hemoglobin, and hematocrit. Therefore, the patient was referred to the Department of Hemato Oncology.

During the evaluation, we started treatment with oral prednisolone 60 mg/day after eliminating the possibility of infection based on the history and laboratory examination results. Blurred vision in both eyes began to improve 3 days after treatment, and the visual acuity of the left eye improved to 20/20. The dose of oral prednisolone was tapered off, and the visual acuity of the right eye improved to 20/40 after 5 days and 20/32 after 2 weeks. Based on the results of a complete blood count and the following findings: increased serum protein and decreased albumin, M-peak findings on serum and urine protein electrophoreses, peripheral blood smear, plasma cell findings on bone marrow biopsy, and increased IgA and free lambda light chain, the patient was diagnosed with multiple myeloma. The bortezomib (Velcade), lenalidomide (Revlimid), and dexamethasone (VRD) regimen was chosen for the treatment of multiple myeloma. Therefore, oral prednisolone was replaced with intravenous dexamethasone. Dexamethasone was administered intravenously for 2 weeks, and the visual acuity of the right eye improved to 20/20 1 month after starting treatment. Subsequently, the patient developed a neutropenic fever. No source of infection was found; however, steroids were discontinued because of the elevation in infectious markers. After 4 weeks of steroid treatment, the visual acuity improved in both eyes, optic disc edema improved, and the optic disc margin became clearer. In addition, the intraretinal edema resolved completely, and the subretinal fluid improved to the extent that only a little remained.

## Discussion

This patient was diagnosed with idiopathic neuroretinitis after eliminating the possibility of infectious causes, and non-infectious causes, including inflammation disorders, malignant hypertension, and so on. The patient improved with steroid treatment, and multiple myeloma was confirmed in the evaluation of hematological abnormalities during treatment. The case of neuroretinitis associated with multiple myeloma is very rare.

Impaired permeability of the optic disc vasculature, and infectious or inflammatory processes have been proposed to be responsible for the pathogenesis of neuroretinitis [4, 5]. Table 1 lists other entities that may cause neuroretinitis [1, 6].

**Table 1:** Differential Diagnosis of Neuroretinitis

Infectious	Noninfectious
Bartonellosis (Bartonella henselae)	Sarcoidosis
Lyme disease	Systemic lupus erythematosus
Syphilis	Behcet disease
Tuberculosis	Polyarteritis nodosa
Actinomycosis	Takayasu’s arteritis
Salmonella	Anterior ischemic optic neuropathy
Influenza	Venous occlusion
Rubella	Systemic hypertension
Measles	Diabetes mellitus
Mumps	Idiopathic intracranial hypertension
Varicella	
Herpes simplex and zoster	
Zika	
Coccidioidomycosis	
Toxoplasmosis	
Toxocariasis	
Diffuse unilateral subacute neuroretinitis	
Leptospirosis	

This process results in optic disc swelling, secondary leakage to the surrounding retina, and serous retinal detachment, followed by the formation of hard exudates at the level of the outer plexiform layer, giving rise to the macular star appearance (often 2–6 weeks after the onset of the initial symptoms). In most cases of neuroretinitis, macular edema, rather than optic disc edema, is largely responsible for vision loss. Hence, the resolution of macular edema and exudates is positively correlated with vision improvement [7]. Classically, disc edema begins to decrease 2 weeks after onset, and by 3 months, most patients experience complete resolution [8].

The idiopathic subtype accounts for nearly half of the cases of neuroretinitis, and consensus on the treatment of this variant is lacking [7]. Idiopathic neuroretinitis can be divided into single isolated and recurrent episodes. Idiopathic neuroretinitis usually occurs once, and most cases demonstrate spontaneous resolution and usually have a favorable prognosis; however, recurrence portends a poorer visual prognosis [9, 10]. Certain clinical features may act as predictors of recurrence in patients with idiopathic neuroretinitis. Greater severity of visual field deficits and other patterns besides central or cecocentral scotoma, preserved visual acuity with a large relative afferent pupil defect, poor recovery of vision, and the absence of prodromal systemic symptoms that usually accompany infectious neuroretinitis has been shown to be predictive factors for recurrence [4].

Depending on the cause, treatment includes steroids, antibiotics, or combination therapy. After excluding an infectious etiology, idiopathic neuroretinitis is treated with steroids during the acute episodes. If the patient is at risk for recurrent idiopathic neuroretinitis, intravitreal steroid injection and oral or intravenous steroids and immunosuppressive agents may be considered for long-term prophylaxis, which reportedly reduces the frequency of recurrence [7, 10]. As the treatment and prognosis vary according to the cause, ophthalmologists should consider the differential diagnosis of optic disc edema with a macular star, i.e., neuroretinitis masqueraders. Given that neuroretinitis is usually unilateral, a bilateral presentation should make the examiner highly suspicious of a neuroretinitis mimic; however, neuroretinitis cannot be completely ruled out because bilateral cases have been reported [1].

It is not uncommon for masqueraders to be mistaken for neuroretinitis. One study found that 35% of patients referred to a neuro-ophthalmology clinic for the evaluation of neuroretinitis had mimic disorders presenting with optic disc edema with a macular star and were more common than neuroretinitis [3]. Newly diagnosed malignant hypertension was the most common masquerader (43%). Other masqueraders include branch retinal vein occlusion (21%), idiopathic intracranial hypertension (IIH) (14%), diabetic papillopathy (14%), and anterior ischemic optic neuropathy (7%). IIH is one of the conditions that we need to differentiate along with malignant hypertension in neuroretinitis, so when we see a patient with suspected neuroretinitis, it is important to rule out IIH by checking for neurologic symptoms, including headache, nausea/vomiting, visual disturbances such as diplopia, transient visual loss, blurred vision, and hearing impairment, which are common in IIH, performing tests such as brain MRI or CT imaging, MRV and MRA to check for vascular abnormalities such as venous stasis in sinuses, aneurysm, thrombus, and lumbar puncture to measure CSF opening pressure and analyze its components [11] and referral to neurology.

In the present case, the patient had multiple myeloma in addition to neuroretinitis. It is not possible to determine the sequence of occurrence, because the exact timing of the onset of multiple myeloma was unknown. Therefore, it was difficult to determine whether multiple myeloma influenced the development of neuroretinitis. Multiple myeloma is a plasma cell disorder that accounts for 13% of all hematological malignancies and 1% of all neoplastic diseases [12]. Ophthalmic manifestations are a common feature of hematological malignancies and can be divided into two groups: those attributable to the disease’s infiltration of the eye and ocular sequelae due to blood abnormalities. Bouazza et al. reported that ocular manifestations were the most frequent in hematological malignancies such as acute myeloid leukemia (76%), non-Hodgkin’s lymphoma (65.2%), and multiple myeloma (57.6%) [13]. Different ocular lesions such as retinal hemorrhage, Roth spots, and dry eye disease have been reported to be the most common manifestations. Dry eye, retinal hemorrhage, subconjunctival hemorrhage, and central retinal vein occlusion occur in multiple myeloma. Other studies reported the development of dry eye, cataracts, and glaucoma [12, 14].

However, none of the several studies cited above have mentioned the association between multiple myeloma and neuroretinitis. Only one study has investigated the association between the neuroretinitis and multiple myeloma treatment regimens. Lennan et al. [15] reported that a 53-year-old man with multiple myeloma developed severe acute bilateral optic neuroretinitis 8 days after chemotherapy with carmustine, procarbazine, cyclophosphamide, and prednisolone. Procarbazine is known to interfere with neurological function. Carmustine has been reported to interfere transiently with vision. These two drugs raise the possibility that one or both combinations may have caused optic neuropathy. However, since this patient developed neuroretinitis before the treatment of multiple myeloma, our case report is the first description of neuroretinitis associated with multiple myeloma.

## Conclusion

Neuroretinitis has various etiologies and neuroretinitis masqueraders are common. Since treatment and prognosis vary depending on the cause, differential diagnosis and careful evaluation are required. It is important to distinguish between infectious and non-infectious etiologies, mostly inflammatory and idiopathic disorders. It is important to determine whether there is a risk of recurrence in idiopathic cases, owing to the poor prognosis. In addition, although neuroretinitis is rarely accompanied by a hematological malignancy, it is important to be mindful of hematological diseases because ophthalmic manifestations are a common feature of these neoplasms and present with lesions occurring in nearly every ocular structure.

## Data Availability

The datasets from the current study can be obtained on request from the corresponding author.
